# Case Report: A Novel Solution to Penile Zipper Injury—The Needle Holder

**DOI:** 10.1100/tsw.2005.39

**Published:** 2005-04-06

**Authors:** Philip A. McCann

**Affiliations:** Department of Emergency Medicine, Cheltenham General Hospital, Sandford Road, Cheltenham, GL53 7AA, UK

**Keywords:** penile trauma, zipper

## Abstract

Penile injuries are relatively uncommon[1]. The crush injury mediated by entrapment of the skin between the teeth and fastener of a zipper mechanism has been described[2,3]. It is seen more commonly in uncircumcised children than adults[4]. A number of treatment methods have been mentioned in the literature[5,6]. An adult case presentation and novel method of management using two small needle holders is illustrated.

## CASE REPORT

A 21-year-old, uncircumcised man presented to the accident and emergency department minor injuries unit out of hours after accidentally trapping the dorsal surface of the penis in the zipper of his jeans. The incident happened whilst fastening the zipper upwards. The patient immediately experienced pain and discomfort in the penis. After a number of attempts to free the penis himself, the gentleman decided to attend the department.

On examination, the gentleman had trapped approximately 20 mm of skin in the zipper mechanism at the midpoint of the body of the penis, in two separate sections, 8 mm each, separated by 2 mm of interdigitating metal teeth. There was only very slight bleeding from the involved skin and minimal oedema.

Initially, the zipper mechanism was cut from the surrounding material. The patient was given entonox for analgesia and sedation. Numerous attempts at removing the zipper with lubricant failed.

It was then decided to try to free the zipper mechanism by cutting the remaining cloth from the teeth of the in the hope that it would serve to loosen the purchase the teeth had on the skin. This also proved useless and by this stage, the patient was becoming increasingly anxious and understandably less cooperative.

The only instruments available at this point were those in a standard suture pack: forceps, scissors, and needle holders. After some consideration, it was decided to try to free the fastener by using two needle holders, placing one on the upper aspect and one opposite on the lower aspect of the fastener. By applying firm pressure and pulling the needle holders in opposite directions, the teeth could be gently separated from the entangled skin as the grasp on the tissues within the mechanism was now relieved. Examination of the underlying skin revealed only a minor abrasion, which was cleaned and dressed. The patient was discharged the same evening.

## DISCUSSION

A number of methods have been described for the management of entrapment of penile skin within a zipper. The most conservative of these is manipulation of the skin from the fastener one tooth at a time[7]. This may be made easier using lubrication[8]; however, even though this method may work, it is not to be pursued too aggressively as it may indeed make the problem worse, by aggravating the tissues and increasing the local oedema[4] .

Initially, this problem was managed in children by circumcision[7]. The most obvious disadvantage of this procedure is that it is clearly only suitable for the uncircumcised patient with an injury to the prepuce. However, this may not be the most palatable option as it necessitates a general anaesthetic in addition to conferring its own operative risks[9,10,11]. It may also be unacceptable for various social/cultural reasons.

In the late 1970s, Flowerdrew et al.[3] described a method that involved using a bone cutter to separate the median bar of the fastener to undo the zipper. The disadvantages of this method are the probable need for general anaesthesia and the availability of the instrument[8], which may not (as in this case) be to hand.

Oosterlinck[6] described using a series of transverse cuts to free the material from around the teeth. This approach was tried in this case (fortuitously, without prior knowledge of the technique), but to no avail as the teeth had too much purchase on the skin.

Review of the literature yielded no previous report of a similar technique. The needle holder method requires only minimal analgesia, basic equipment, and is simple to perform. Therefore, it may provide a suitable straightforward alternative in the management of this problematic injury.

## References

1. Mydlo, J.H., Harris, F.C., and Brown, J.G. (2002) Blunt, penetrating and ischaemic injuries to the penis. *J. Urol*. **168(4 Pt 1),** 1433–1435.
2. Saraf, P. and Rabinowitz, R. (1982) Zipper injury to the foreskin. *Am. J. Dis. Child* **136(6),** 557–558.
3. Flowerdrew, R., Fishmann, I.J., and Churchill, B.M. (1977) Management of penile zipper injury. *J. Urol*. **117(5),** 671.
4. Yip, A., Ng, S.K., Wong, W.C., Li, M.K., and Lam, K.H. (1989) Injury to the prepuce. *Br. J. Urol*. **63(5),** 535–538.
5. Nolan, J.F., Stillwell, T.J., and Sands, J.P., Jr. (1990) Acute management of zipper-entrapped penis. *J. Emerg. Med*. **8(3),** 305–307.
6. Oosterlinck, W. (1977) Unbloody management of the penile zipper injury. *J. Urol*. **117(5),** 671.
7. Watson, C.C. (1971) Zipper injuries. *Clin. Pediatr. (Phila.)* **10(3),** 188.
8. Kanegaye, J.T. and Schonfeld, N. (1993) Penile zipper entrapment: a simple and less threatening approach using mineral oil. *Pediatr. Emerg. Care* **9(2),** 90–91.
9. Wiswell, T.E. and Geschke, D.W. (1989) Risks from circumcision during the first month of life compared with those from uncircumcised boys. *Pediatrics* **83(6),** 1011–1015.
10. Ozkan, S. and Gurpinar, T. (1997) A serious circumcision complication: penile shaft amputation and a new reattachment technique with a successful outcome. *J. Urol*. **158(8),** 1946–1947.
11. Gearhart, J.P. and Rock, J.A. (1989) Total ablation of the penis after circumcision with electrocautery: a method of management and long-term follow-up. *J. Urol*. **142(3),** 799–801.
